# A Homeodomain-Containing Transcriptional Factor *Po*Htf1 Regulated the Development and Cellulase Expression in *Penicillium oxalicum*

**DOI:** 10.3389/fmicb.2021.671089

**Published:** 2021-06-10

**Authors:** Hao Guo, Gen Xu, Ruimei Wu, Zhigang Li, Mengdi Yan, Zhilei Jia, Zhonghai Li, Mei Chen, Xiaoming Bao, Yinbo Qu

**Affiliations:** ^1^State Key Laboratory of Biobased Material and Green Papermaking, Shandong Academy of Sciences, Qilu University of Technology, Jinan, China; ^2^School of Bioengineering, Shandong Academy of Sciences, Qilu University of Technology, Jinan, China; ^3^Shandong Provincial Key Laboratory of Microbial Engineering, Shandong Academy of Sciences, Qilu University of Technology, Jinan, China; ^4^Haixia Institute of Science and Technology, Fujian Agriculture and Forestry University, Fuzhou, China; ^5^State Key Laboratory of Microbial Technology, School of Life Sciences, National Glycoengineering Research Center, Shandong University, Qingdao, China

**Keywords:** homeodomain-containing transcription factors, development, conidiation, cellulase, *Penicillium oxalicum*

## Abstract

Homeodomain-containing transcription factors (Htfs) play important roles in animals, fungi, and plants during some developmental processes. Here, a homeodomain-containing transcription factor *Po*Htf1 was functionally characterized in the cellulase-producing fungi *Penicillium oxalicum* 114-2. *Po*Htf1 was shown to participate in colony growth and conidiation through regulating the expression of its downstream transcription factor BrlA, the key regulator of conidiation in *P. oxalicum* 114-2. Additionally, *Po*Htf1 inhibited the expression of the major cellulase genes by coordinated regulation of cellulolytic regulators CreA, AmyR, ClrB, and XlnR. Furthermore, transcriptome analysis showed that *Po*Htf1 participated in the secondary metabolism including the pathway synthesizing conidial yellow pigment. These data show that *Po*Htf1 mediates the complex transcriptional-regulatory network cascade between developmental processes and cellulolytic gene expression in *P. oxalicum* 114-2. Our results should assist the development of strategies for the metabolic engineering of mutants for applications in the enzymatic hydrolysis for biochemical production.

## Introduction

*Penicillium oxalicum* 114-2 produces diverse lignocellulolytic enzymes, which have been widely used in the effective degradation of agricultural biomass. In the research for hyper-production of cellulases, *P. oxalicum* 114-2 has been used as a model fungus to understand the cellulase regulation systems. A single-gene disruption library of 470 transcription factors has been constructed, and 20 main transcription factors that play putative roles in the activation or repression of cellulase synthesis were identified (Li et al., 2015). PDE_07199 is a cellulase transcription factor involved in both the regulation of cellulase expression and developmental process of *P. oxalicum* 114-2. However, its functional mechanism has not been studied systematically in *P. oxalicum* 114-2.

PDE_07199 amino acid sequence analysis using the Simple Modular Architecture Research Tool (SMART) (Letunic et al., 2021) showed the presence of a homeodomain. Homeodomain-containing proteins have been proved to function as transcription factors and play important roles in animals, fungi, and plants during some developmental processes, such as development and differentiation (Burglin, 2011; Miksiunas et al., 2020). In fungi, homeodomain transcriptional factors (Htfs) play a crucial role in regulating developmental processes. *pah1* from *Podospora anserina* was the first homeobox gene identified in filamentous ascomycetes and was considered to be a repressor of genes involved in the conidiation process. Moreover, *pah1* was also involved in hyphal branching and possibly in the development of female organs (Arnaise et al., 2001). In *Neurospora crassa*, the homolog of *pah1*, *kal-1*, was uncovered as an important regulator of asexual growth and development. *kal-1* mutation lead to substantial changes in colony morphology and conidial development (Colot et al., 2006). In the ascomycete fungus *Magnaporthe oryzae*, eight Htfs were characterized, each of which functions as a stage-specific regulator for conidial shape, hyphal growth, conidiation, appressorium development, and invasive growth during *M. oryzae* development (Kim et al., 2009; Liu et al., 2010). Zheng et al. (2012) reported that a conserved Htf1 is required for phialide development and conidiogenesis in *Fusarium* species. Moreover, Htfs are also involved in fruiting body development in several members of mushroom forming fungi, including *Schizophyllum commune* and *Volvariella volvacea* straw mushroom (Pelkmans et al., 2017; Wang et al., 2019). *Aspergillus nidulans* is a model filamentous fungus that is commonly used for understanding fungal development. Several transcription factors are involved in conidiation in *A. nidulans* including BrlA, FluG, and FlbA-E (Lee and Adams, 1996; Emri et al., 2005; Kwon et al., 2010a, b; Arratia-Quijada et al., 2012; Chang et al., 2012; Oiartzabal-Arano et al., 2015). Recently, two homeodomain proteins, HbxA and HbxB, were functionally characterized in *A. nidulans*. And the two proteins play crucial roles in the conidiophore production and secondary metabolism of *A. nidulans* (Son et al., 2020).

Previously reported Htfs play regulatory roles during different developmental stages in fungi. In *P. oxalicum* 114-2, a homeodomain-containing protein called PDE_07199 (*Po*Htf1) was identified. *Po*Htf1 is both crucial for development and in the regulation of cellulase expression. Our results showed that *Po*Htf1 mediates the complex transcriptional-regulatory network cascade between developmental processes and cellulolytic gene expression.

## Materials and Methods

### Strains and Culture Conditions

The *P. oxalicum* 114-2 (CGMCC 5302) wild-type (WT) strain was stored in our laboratory. Spores of *P. oxalicum* strains were cultured on wheat bran agar medium at 30°C for 4 days and harvested using sterile water. The final concentration was over 10^10^ spores/mL. For cellulase production, 10^8^/mL spores were inoculated in Vogel’s salts liquid medium containing 2% glucose and incubated at 30°C with shaking at 200 rpm for 24 h. Then, the mycelia were collected by vacuum pump filtration. Exactly 0.5 g of mycelia was transferred into 50 mL of cellulase production medium containing Vogel’s salts liquid medium, 1% wheat bran, and 1% microcrystalline cellulose. Cellulase production was performed in a 300 mL flask at 30°C with shaking at 200 rpm.

### Construction of *Pohtf1* Deletion and Complement Strains

*Pohtf1* gene deletion was performed using the homologous recombination method. The up- and down-stream homologous flanks of *Pohtf1* were amplified from *P. oxalicum* 114-2 genomic DNA with primers 7199-F1/7199-ptraR and 7199-ptraF/7199-R1. The selected marker gene, *ptrA*, was amplified from the pME2892 plasmid with ptrA-F1/ptrA-R1 primers. The three fragments were fused using double-joint PCR (Yu et al., 2004), and the full-length deletion cassette was amplified by primer 7199-F2/7199-R2. The deletion cassette was transformed into *P. oxalicum* 114-2 using the PEG-mediated method (Li et al., 2010) to obtain the *Pohtf1* gene knock-out strain Δ*Pohtf1*.

For *Pohtf1* gene complementation, the integrated expression cassette was amplified from *P. oxalicum* 114-2 genomic DNA with primers 7199-hphF/7199-hphR. Hygromycin resistance gene (*hph*) was amplified from the pSilent-1 plasmid using primers hph-F/hph-R. The two fragments were fused, and the complement cassette was amplified using primers 7199-F4/7199-R4. The cassette was transformed into strain Δ*Pohtf1* as described above, and the complement strain C*Pohtf1* was constructed.

Transformants were analyzed using full-length amplifying primers. All primers used in the construction of strains Δ*Pohtf1* and C*Pohtf1* are listed in Supplementary Table 1.

### Transcriptome Analysis

For transcriptome analysis, 10^8^/mL spores were inoculated in Vogel’s salts liquid medium containing 2% glucose and incubated at 30°C with shaking at 200 rpm for 24 h. Then, the mycelia were collected by vacuum pump filtration and transferred into Vogel’s salts liquid medium lacking a carbon source. After 2 h incubation, the mycelia were collected and 0.5 g of mycelia were transferred into 50 mL of Vogel’s salts liquid medium including 2% microcrystalline cellulose and incubated at 30°C with shaking at 200 rpm for 4 h. The RNA of *P. oxalicum* 114-2 and Δ*Pohtf*1 was extracted using TRIzol reagent (Invitrogen, United States) according to the manufacturer’s protocol. Digital gene expression profiling experiments, based on RNA-Seq, were performed using the Illumina HiSeq 2000 System (Beijing Genomics Institute, China). Gene expression levels were normalized to reads per kb per million reads (RPKM). Significantly differentially expressed genes were filtered with combined thresholds using a false discovery rate (FDR) ≤ 0.001 and fold change ≥ 2. The raw RNA-Seq data were deposited in the National Center for Biotechnology Information (NCBI) Gene Expression Omnibus (GEO) with the series reference number GSE160881.

### Phenotype Analysis

Phenotype analysis was performed on Vogel’s medium plates containing 2% glucose or 1% cellulose and on 10% wheat bran medium plates (Li et al., 2015). Exactly 1 μL of fresh spores with concentration of 10^8^ spores/mL was inoculated into the center of the plates and incubated for 3 days at 30°C.

Spore production capacities of *P. oxalicum* 114-2 and its mutants were measured on Vogel’s medium plates containing 2% glucose. Exactly 250 μL of fresh spores with concentration of 10^8^ spores/mL was spread across the plates. The plates were incubated at 30°C, and the spore numbers in a fixed area were counted at 24, 30, 36, 48, 60, and 72 h.

### Enzyme Activities Assay

For enzyme activities assay, strains *P. oxalicum* 114-2, Δ*Pohtf*1 and C*Pohtf*1 were fermented in the cellulase production medium under the conditions described above for 6 days. Fermentation broths were sampled and measured every 24 h from day 3 to day 6. Whatman^TM^ 1 filter papers, sodium carboxymethyl cellulose (CMC-Na, Sigma), *p*-nitrophenyl-β-D-cellobioside (*p*NPC, Sigma), and *p*-nitrophenyl-β-D-glucopyranoside (*p*NPG, Sigma) were used as substrates for filter paper activity (FPA), endoglucanase, cellobiohydrolase, and beta-glucosidase activities assays, respectively (Chen et al., 2013). One unit of enzyme activity was defined as the amount of enzyme required to produce 1 μmol glucose or *p*-nitrophenyl (*p*NP, Sigma) per minute under the assayed conditions.

### Real-Time Quantitative PCR Analysis

For Real-time quantitative PCR (qRT-PCR), 0.5 g preincubated mycelia of the three strains was induced in 50 mL of cellulase production medium for 4 h. Then, the mycelia were collected for RNA extraction as previously described (Li et al., 2015). The synthesis of cDNA and qRT-PCR reaction were performed using the PrimeScript^TM^ RT reagent kit with gDNA Eraser (Perfect Real Time) and TB Green^®^ Premix Ex Taq^TM^ II (Tli RNaseH Plus) (TAKARA) following the manufactures protocols. qRT-PCR reaction procedure was performed on the LightCycler^®^480 System (Roche) with cycling conditions of: 95°C for 2 min, and 40 cycles of 95°C for 10 s, and 61°C for 30 s. The melting curves were measured with a temperature gradient of 0.1°C per second from 65°C to 95°C. The expression levels of all genes were calculated using the method of relative quantification using *actin* as the reference gene. Primers used for qRT-PCR are listed in Supplementary Table 2. Three biological replicates were performed for all reactions.

## Results

### Sequence and Phylogenetic Analysis of *Po*Htf1

**FIGURE 1:**
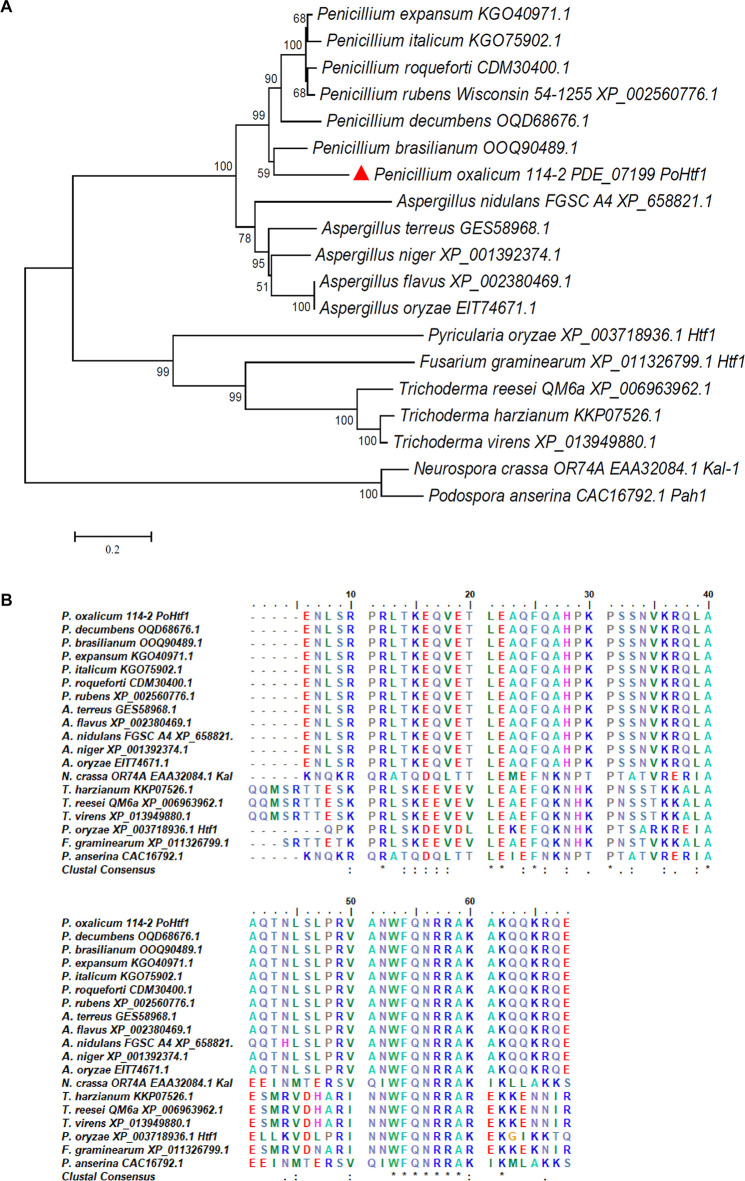
Analysis of the complete and homeodomain sequences of Htfs homologs. **(A)** Phylogenetic analysis of *Po*Htf1 homologs. The complete sequences of Htfs from *Penicillium* sp., *Aspergillus* sp., *Trichoderma* sp., *Neurospora crassa, Fusarium graminearum, Pyricularia oryzae*, and *Podospora anserina* were downloaded from NCBI. The phylogenetic tree was generated by MEGA 5.0 software using the neighbor-joining (NJ) method. **(B)** Sequence alignment of the Htfs homolog homeodomains. The alignment was performed by ClustalW Multiple Alignment function in the Bioedit tool. The consensus residues are labeled by asterisk (*).

The *Po*Htf1 amino acid sequence was analyzed by SMART. A homeodomain of 63 amino acids (amino acid 67–129) was identified in the *Po*Htf1 sequence. Homeodomains proteins performs its regulatory function by binding DNA through the helix-turn-helix structure (Dorn et al., 1994). The *Po*Htf1 homologs of *Po*Htf1 were identified using the Basic Local Alignment Search Tool (Blast) in the National Center of Biotechnology Information (NCBI) database. The *Po*Htf1 amino acid sequence was closely related to those of other *Penicillium* species (Figure 1A). *Po*Htf1 shared the highest identity of 67.37% with the *P. brasilianum* homolog (OOQ90489.1), and shared identities of 41.51 and 45.35% with the *A. nidulans* homolog, HbxA (AN1217.2, XP_658821.1) and *Trichoderma reesei* QM6a homolog (XP_006963962.1), respectively. However, *Po*Htf1 was distantly related to several reported Htfs that function in developmental processes in filamentous fungi. *Po*Htf1 only shared identities of 17 and 18% with the first identified Htf in *P. anserine*, Pah1 (CAC16792.1) and its homolog in *N. crassa* OR74a, Kal-1 (NCU03593, EAA32084.1), respectively. Htfs from *M. oryzae* (MGG_00184, XP_003718936) and *F. graminearum* (FGSG_07097, XP_011326799.1), which are involved in hyphal growth and conidiogenesis (Liu et al., 2010; Zheng et al., 2012), shared 40.94 and 22.00% identities, respectively, with *Po*Htf1.

The homeodomain sequences of the Htf homologs were aligned by ClustalW Multiple Alignment. The Htf homeodomains from *Penicillium* and *Aspergillus* species were highly conserved. However, the *Po*Htf1 homeodomain shared a low level of identity with homeodomains from other strains (Figure 1B). These data indicate that *Po*Htf1 probably has conserved functions in developmental processes, like those in *Aspergillus* species. However, *Po*Htf1 might execute its regulatory functions differently than related proteins do in *F. graminearum* and *M. oryzae*.

### Comparative Transcriptome Analysis

**FIGURE 2:**
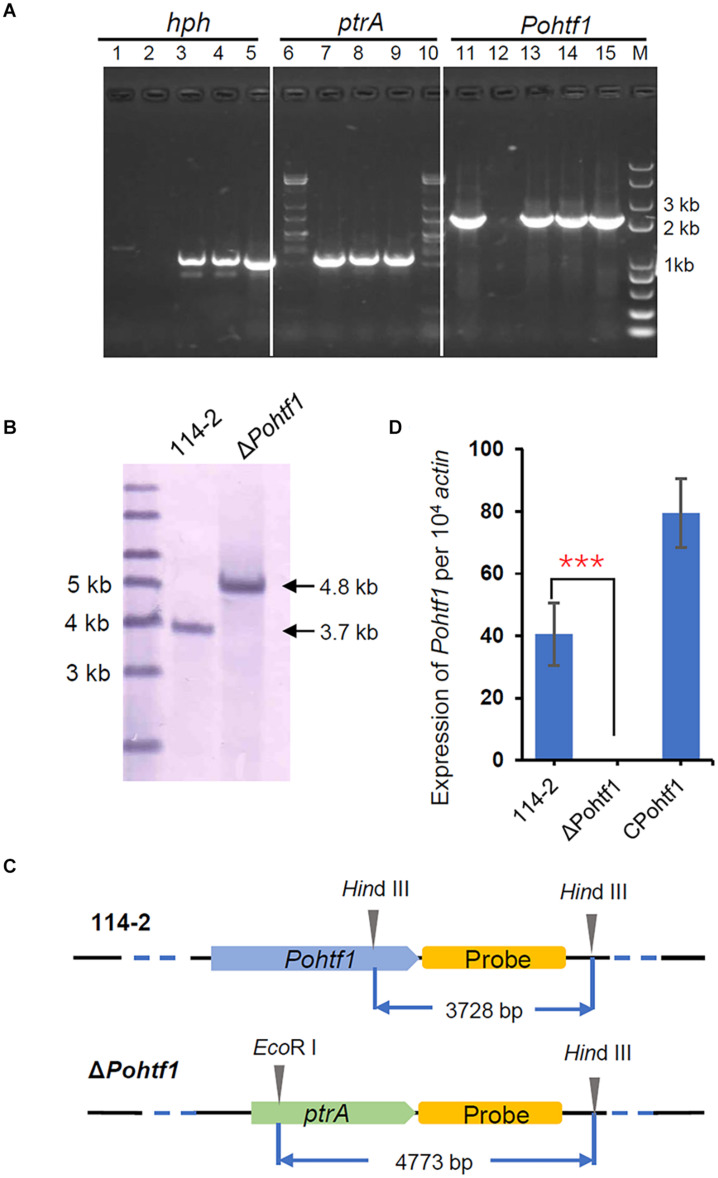
Deletion and verification of *Po*Htf1. **(A)** PCR analysis of *Pohtf1* deletion and complement. The confirmation of *Pohtf1* deletion and complement was performed using three pairs of primers: primers inside the selected marker genes *hph* (hph-YZ-F/R) (lane 1–5) and *ptrA* (ptrA-YZ-F/R) (lane 6–10) and primers amplifying the target gene *Pohtf1* (7199-hphF/7199-hphR) (lane 11–15). Primer sequences are listed in Supplementary Table 1. Lane 1, 6, 11 are the results of the WT strain *P. oxalicum* 114-2. Lane 2, 7, 12 are the results of *Pohtf1*-deletion strain. Lane 3–4, 8–9, and 13–14 are the results of two *Pohtf1*-complement strains. **(B)** Southern blot analysis of Δ*Pohtf1*. After deletion of *Pohtf1*, a 4.8 kb-length fragment was hybridized in the Δ*Pohtf1* genome by Southern blot, while the fragment length in *P. oxalicum* 114-2 was 3.7 kb. **(C)** Southern blot strategy. A 2 kb-length fragment downstream of the target gene was amplified as the hybridization probe. *P. oxalicum* 114-2 genomic DNA was digested by a single restriction endonuclease, *Hin*dIII, and a 3.7 kb-length fragment containing the complete probe sequence was generated. The Δ*Pohtf1* genomic DNA was double digested with *Hin*dIII and *Eco*RI to generate a 4.8 kb-length fragment. **(D)** qRT-PCR analysis of *Pohtf1* expression levels in the mutant strains. *Pohtf1* expression levels in *P. oxalicum* 114-2, Δ*Pohtf1*, and C*Pohtf1* were tested using primers RT-7199-F/R inside *Pohtf1* (Supplementary Table 2).

To investigate the functions of *Po*Htf1 at the whole genome level, *Po*Htf1 was deleted in the *P. oxalicum* 114-2 strain using homologous recombination. *Po*Htf1 deletion was verified by PCR, Southern blot, and qRT-PCR (Figure 2). The effects of *Po*Htf1 deletion were analyzed by comparing the transcriptomes of *P. oxalicum* 114-2 and Δ*Pohtf1* using RNA-Seq (Li et al., 2015). After *Po*Htf1 deletion, there were 158 up-regulated and 237 down-regulated genes. Moreover, the differentially expressed genes were mainly involved in starch and sucrose metabolism (11 genes), glycolysis (8 genes), and tyrosine metabolism (6 genes) pathways (Supplementary Figure 1).

**FIGURE 3:**
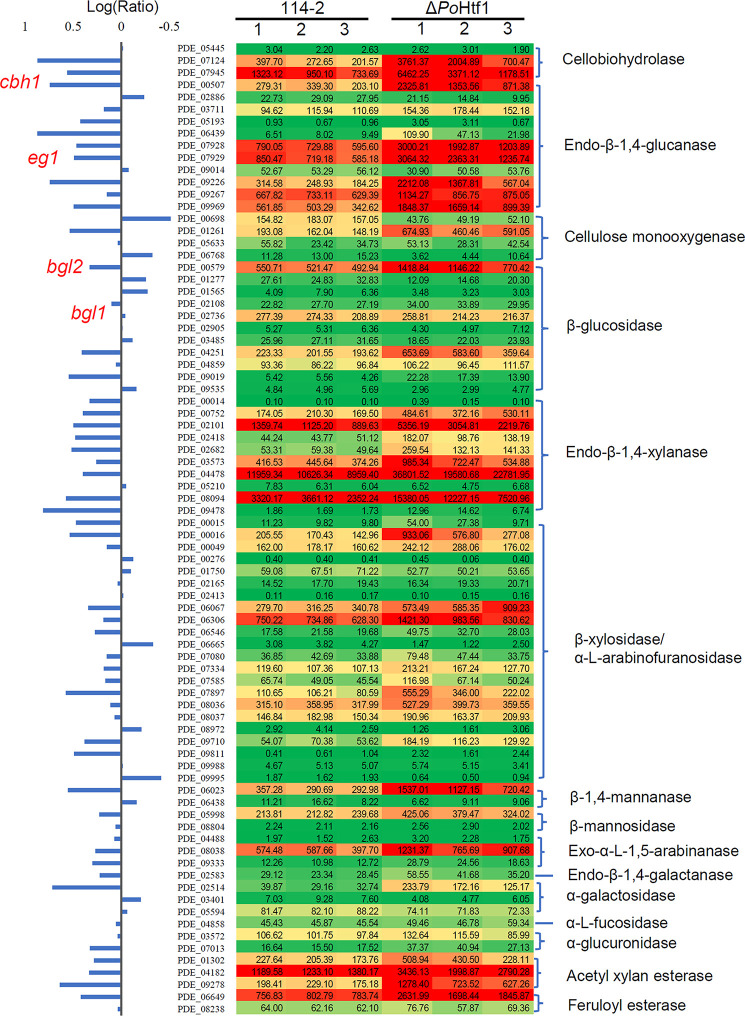
Expression profile of 80 annotated cellulose hydrolyase genes in *P. oxalicum* 114-2 and Δ*Pohtf1*. The expression levels (RPKM) of the 80 genes are labeled by green-yellow-red color scales in excel. The minimum value is 0, the median is 100, and the maximum value is 36,801.

Given the potential of *Po*Htf1 to regulate cellulases, the expression levels of 80 annotated cellulolytic genes were analyzed. The results showed that the expression levels of most of cellulase and hemicellulase genes were substantially up-regulated in Δ*Pohtf1* (Figure 3). Among these genes, the major cellobiohydrolase gene *cbh1*, endo-β-1,4-glucanase gene *eg1* and intracellular β-glucosidase gene *bgl2* were up-regulated in Δ*Pohtf1*, with expression levels nearly 3. 7-, 3. 1-, and 2.1-fold higher, respectively, than those in *P. oxalicum* 114-2. However, *bgl1* which encodes the major extracellular constitutive beta-glucosidase was not differentially expressed between strains 114-2 and Δ*Pohtf1*.

Moreover, secondary metabolism was also influenced by the absence of *Po*Htf1. Several secondary metabolic gene clusters were substantially up- or down-regulated in Δ*Pohtf1* (Supplementary Table 3). Comparative transcriptome results showed that two predicted gene clusters that synthesized oxaline (Newmister et al., 2016) and aspyridones (Klejnstrup et al., 2012) were down-regulated. Similarly, expression of the gene cluster generating conidial yellow pigment (Viggiano et al., 2018) also decreased, possibly altering the Δ*Pohtf1* conidium and colony phenotype. Furthermore, five unknown secondary metabolic gene clusters were down-regulated, and two unknown gene clusters were up-regulated in Δ*Pohtf1* (Supplementary Table 3).

The comparative transcriptome results indicate that *Po*Htf1 is not only crucial in development, but also for the expression of cellulose hydrolyases and secondary metabolism in *P. oxalicum* 114-2.

### *Po*Htf1 Functions in Development and Conidiation in *Penicillium oxalicum* 114-2

**FIGURE 4:**
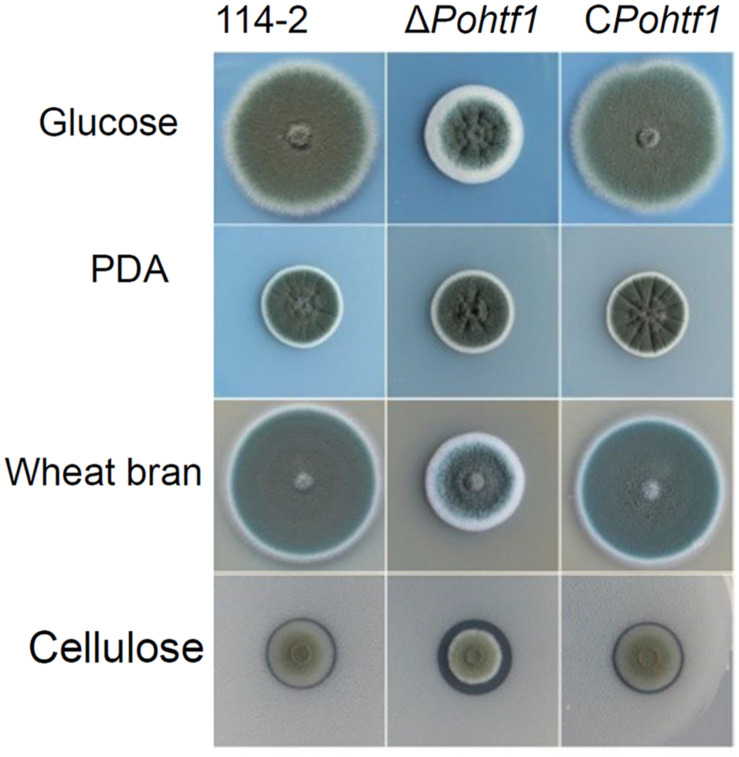
Colony phenotypes of strains *P. oxalicum* 114-2, Δ*Pohtf1* and C*Pohtf1*. Phenotype analysis was performed on Vogel’s medium plates containing 2% glucose or 1% cellulose, PDA plates and 10% wheat bran medium plates. The plates were incubated for 3 days at 30°C.

The influence of *Po*Htf1 on *P. oxalicum* 114-2 development was assessed by measuring colony phenotypes and conidiation. The phenotypes on glucose plates showed that *Po*Htf1 deletion restrained colony growth and conidiation (Figure 4), and Δ*Pohtf1* colonies were much smaller than those of the *P. oxalicum* 114-2 parental strain. The hydrolysis halo of Δ*Pohtf1* on cellulose plates was much more obvious than that of *P. oxalicum* 114-2, indicating enhanced cellulase production in Δ*Pohtf1*.

**FIGURE 5:**
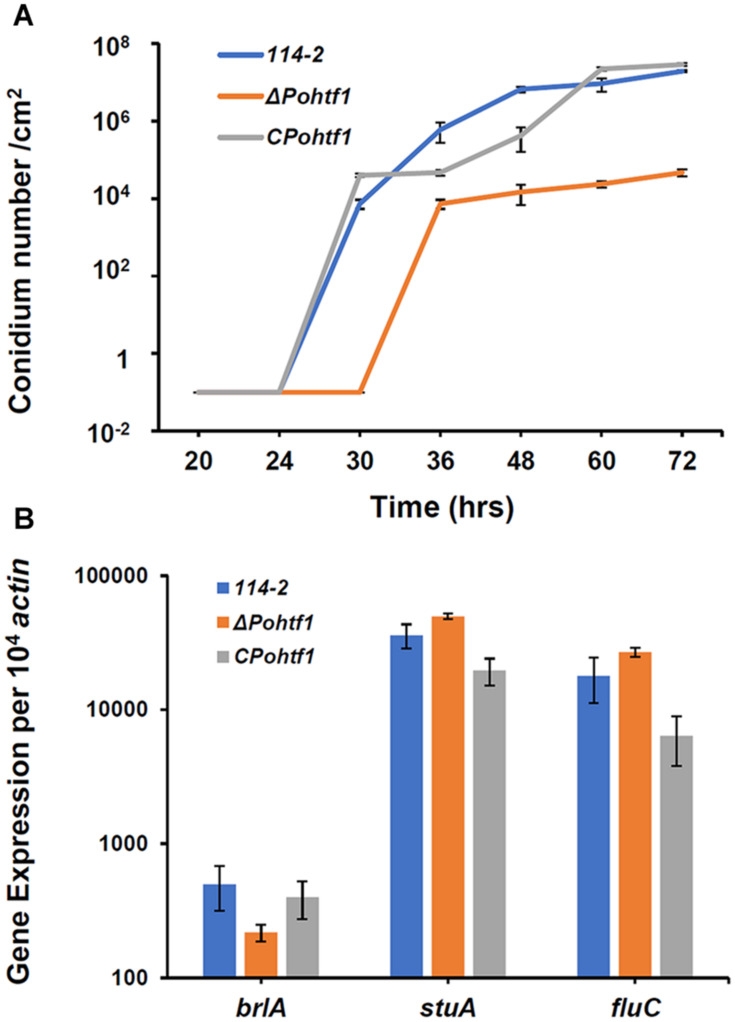
The effects of *Po*Htf1 on conidiation. **(A)** The conidiation curve of *P. oxalicum* 114-2, Δ*Pohtf1* and C*Pohtf1* on glucose plates. The plates were incubated at 30°C, and the spore numbers in a fixed area were counted at 24, 30, 36, 48, 60, and 72 h. **(B)** Expression of the major asexual development transcriptional regulators. All strains were incubated in the cellulase production medium for 4 h. The expression levels of *brlA*, *stuA* and *fluC* were measured by qRT-PCR.

The conidiation of Δ*Pohtf1* was delayed and visibly restricted on glucose plates. The conidium of Δ*Pohtf1* was detected after incubation for 36 h on glucose medium plates, which is 6 h later than that of the parental strain *P. oxalicum* 114-2 (Figure 5A). Furthermore, Δ*Pohtf1* produced fewer conidium, by nearly three orders of magnitude, than *P. oxalicum* 114-2 did after incubation for 72 h on glucose medium plates.

To investigate the mechanism by which *Po*Htf1 regulates conidiation, we analyzed how *Po*Htf1 affects the genes that function in the development and conidiation of *P. oxalicum* 114-2. In *P. oxalicum* 114-2, three key transcriptional regulators of asexual development have been identified, BrlA, FlbC, and StuA. These proteins regulate the expression levels of pigmentation-related and spore wall protein-related genes (Qin et al., 2013; Yao et al., 2016; Li et al., 2019). Transcription levels of *brlA*, *flbC*, and *stuA* were analyzed in all strains by RT-PCR. After deletion of *Pohtf1*, the expression levels of *brlA* in Δ*Pohtf1* were 44% of that in *P. oxalicum* 114-2 (Figure 5B). The expression levels of *flbC* and *stuA* were almost unchanged. BrlA is crucial in conidiation, and deletion of *brlA* completely blocks the conidiation of *P. oxalicum* 114-2 (Qin et al., 2013). Therefore, we assumed that *Po*Htf1 functions in conidiation by regulating the transcription levels of the key regulator, BrlA.

### *Po*Htf1 Deletion Promotes the Expression of Cellulases

**FIGURE 6:**
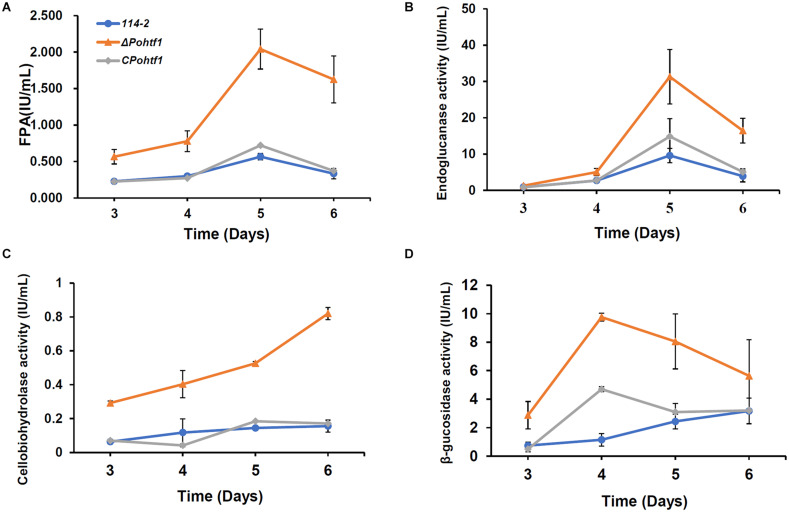
Analysis of the cellulase activities of *P. oxalicum* 114-2, Δ*Pohtf1* and C*Pohtf1*. **(A)** Filter Paper activity (FPA), **(B)** Endoglucanase, **(C)** Cellobiohydrolase and **(D)** β-glucosidase activities of strains *P. oxalicum* 114-2, Δ*Pohtf1* and C*Pohtf1* were assessed. All the strains were sampled and measured every 24 h from day 3 to day 6.

Besides regulating colonial growth and conidiation, *Po*Htf1 also inhibits cellulase expression in *P. oxalicum* 114-2. The effect *Po*Htf1 on the expression of cellulases was determined by assessing the activities toward filter paper (indicating overall cellulase activity), CMC-Na (endoglucanase), *p*NPC (cellobiohydrolase activity), and *p*NPG (β-glucosidase). Δ*Pohtf1* showed the highest FPA after fermentation for 5 days, increasing from 0.6 IU/mL in *P. oxalicum* 114-2 to 2.0 IU/mL in Δ*Pohtf1* (Figure 6A). Similarly, the endoglucanase activity increased to nearly fivefold of that of *P. oxalicum* 114-2 on the 5th day (Figure 6B). The cellobiohydrolase activity continued to increase on the 6th day and was almost fivefold of that of *P. oxalicum* 114-2 (Figure 6C). The maximum β-glucosidase activity was observed on the 4th day, and it increased to 9.8 IU/mL in Δ*Pohtf1* from 1.1 IU/mL in *P. oxalicum* 114-2 (Figure 6D). Together, the activity analysis showed that the absence of *Pohtf1* promoted cellulase production.

**FIGURE 7:**
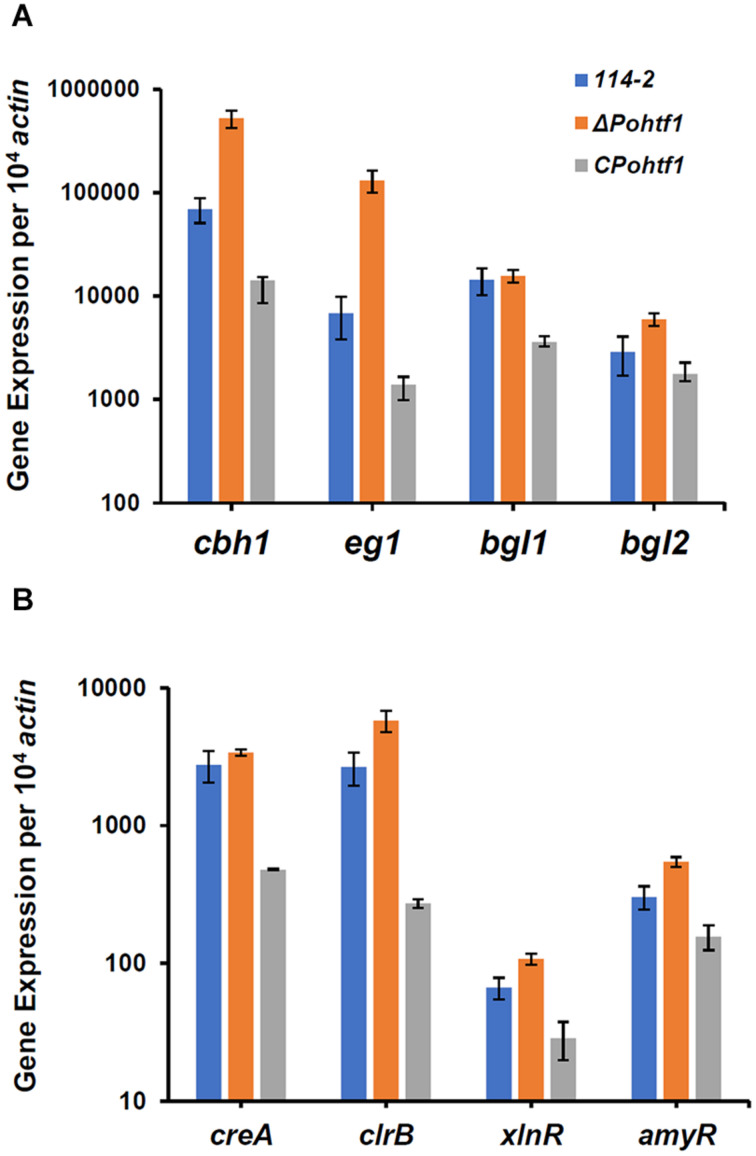
qRT-PCR analysis of the major cellulases and transcriptional regulators in *P. oxalicum* 114-2, Δ*Pohtf1* and C*Pohtf1*. **(A)** Expression levels of the four cellulase genes, *cbh1*, *eg1*, *bgl1*, and *bgl2*; **(B)** Expression levels of the four transcriptional regulator genes, *creA*, *clrB*, *xlnR*, and *amyR*. All strains were incubated in the cellulase production medium for 4 h. The gene expression levels were measured by qRT-PCR.

To investigate the mechanism of *Po*Htf1 in regulating cellulase expression, we analyzed the transcriptional levels of the main cellulase genes and regulators. Mycelium of all the strains were induced in cellulase production medium for 4 h. The expression levels of four major cellulase genes, *cbh1*, *eg1*, *bgl1*, and *bgl2*, were measured by qRT-PCR. In Δ*Pohtf1*, the expression levels of the major cellobiohydrolase and endoglucanase genes *cbh1* and *eg1* were 7.5- and 19.2-fold higher, respectively, than those observed in *P. oxalicum* 114-2 (Figure 7A). This is consistent with the observed increased FPA, cellobiohydrolase, and endoglucanase activities in Δ*Pohtf1*. Additionally, the expression of major intracellular β-glucosidase gene *bgl2* was twofold higher than that observed in *P. oxalicum* 114-2, while the expression level of the main extracellular β-glucosidase gene *bgl1* of Δ*Pohtf1* did not increase. These results are consistent with the RNA-Seq comparison of strains Δ*Pohtf1* and 114-2.

To investigate whether the up-regulation of cellulase genes following *Po*Htf1 deletion was occurred through regulating cellulolytic transcriptional factors, we measured the expression levels of CreA, AmyR, ClrB, and XlnR in Δ*Pohtf1* (Figure 7B). CreA is the most important cellulase transcription repressor and can seriously limit cellulase expression under inducing conditions in *P. oxalicum* 114-2. AmyR is the major activator of amylases and can repress the expression of cellulases. ClrB and XlnR are the major activators of cellulases and xylanases, respectively, and both can markedly enhance the expression of cellulases (Li et al., 2015). Our results showed that the expression of the two repressors CreA and AmyR and two activators ClrB and XlnR were enhanced 23, 80, 117, and 62%, respectively, by the deletion of *Pohtf1* when compared with the expression levels in *P. oxalicum* 114-2 (Figure 7B). These results indicate that regulation of *Po*Htf1 toward the four transcription factors was not specific, and that the inhibiting effect of *Po*Htf1 on cellulases was the result of coordinated regulation of the transcription factors.

## Discussion

In this study, we identified a conserved homeodomain-containing transcriptional regulator *Po*Htf1, an important regulator of genes involved in colonial growth and conidiation in *P. oxalicum* 114-2. After deletion of *Po*Htf1, colony growth and conidiation were substantially limited (Figures 4, 5). In *P. oxalicum* 114-2, there are five other *Po*Htfs (PDE_00454, PDE_03945, PDE_03970, PDE_04931, and PDE_05741) annotated. However, only *Po*Htf1 displayed developmental regulatory function (Li et al., 2015). This is mainly because *Po*Htf1 is the major *Po*Htf in *P. oxalicum* 114-2, as its transcription level is much higher than that of other *Po*Htfs (Supplementary Figure 2). After deletion of *Po*Htf1, the other five *Po*Htfs could not compensate for function because their transcription levels were almost unchanged in Δ*Pohtf1* (Supplementary Figure 2). Furthermore, there were only five highly conserved residues in the homeodomain sequences of the six *Po*Htfs (Supplementary Figure 3). This suggested that the six *Po*Htfs might participate in different developmental processes in *P. oxalicum* 114-2.

Conidiation is the most common reproductive mode of many filamentous fungi. Htf homologs are necessary for conidiation in other fungi including *A. nidulans, M. oryzae*, and *F. graminearum* (Liu et al., 2010; Zheng et al., 2012; Son et al., 2020). In *M. oryzae*, HTF1 is the essential transcription factor for conidiogenesis, and the *htf1* mutant strain failed to differentiate conidia (Liu et al., 2010). Some evidence revealed that the G protein-cAMP signaling pathway is implicated in conidiation through regulating HTF1 expression. Mutation of trimeric G-protein β subunit (*mgb1*) and cAMP-dependent protein kinase gene (*cpkA*) significantly downregulated HTF1 expression (Zhou et al., 2009). However, HTF1 is not a direct regulator of conidiation. Transcription factors ACR1 and CON7 participate in conidiation and conidium morphology in *M. oryzae* (Lau and Hamer, 1998; Odenbach et al., 2007). And Htf1 may regulate conidiation by interacting with ACR1, as ACR1 is significantly regulated by HTF1 (Liu et al., 2010). In *A. nidulans*, HbxA and HbxB were identified to participate in conidia production in *A. nidulans* (Son et al., 2020). BrlA is a key regulator of conidiation (Adams et al., 1988, 1998), and HbxA effected conidiation by regulating *brlA* expression (Son et al., 2020).

The *Po*Htf1 homeodomain sequence is highly conserved with that of HbxA in *A. nidulans* (Figure 1B), suggesting that *Po*Htf1 and HbxA have identical conidiation regulation mechanisms. There are three key transcriptional regulators of asexual development, BrlA, FlbC, and StuA, which are involved in conidiation and conidium morphology in *P. oxalicum* 114-2 (Qin et al., 2013; Yao et al., 2016; Li et al., 2019). BrlA is the key regulator of conidiation, and deletion of *brlA* completely blocks conidiation. *Po*Htf1 deletion significantly represses *brlA* expression (Figure 5B), indicating that *Po*Htf1 might be the upstream regulator of BrlA. FlbC is also an upstream regulator of BrlA, and *flbC* deletion leads to significant downregulation of *brlA* and impaired conidiation (Yao et al., 2016). However, *Po*Htf1 do not appear to interact with FlbC or StuA, as deletion of *Po*Htf1 barely caused any effect on the expression levels of FlbC and StuA (Figure 5B). Therefore, we assumed that *Po*Htf1 regulated conidiation *via* BrlA, similar to the regulation pattern observed in *A. nidulans* (Son et al., 2020).

*Penicillium oxalicum* produces diverse cellulolytic enzymes, which are coordinately regulated by the combinations of many transcription factors. Regulators of asexual development, such as BrlA and FlbC, are involved in the regulation of cellulase expression (Qin et al., 2013; Yao et al., 2016). Deletion of *brlA* upregulates the expression of most of the major cellulases (Qin et al., 2013). While the absence of *flbC* reduces cellulase and hemicellulase production (Yao et al., 2016). In this study, deletion of *Po*Htf1 also increased cellulase production in *P. oxalicum* 114-2 (Figure 6). These results suggest that there is some cross-regulation between cellulase expression and asexual development. To investigate the mechanism of *Po*Htf1 in regulating the expression of cellulases, we determined the expression levels of four major identified transcription factors involved in regulating cellulase expression, CreA, AmyR, ClrB, and XlnR, in Δ*Pohtf1*. CreA is the most important transcription repressor of cellulases in *P. oxalicum* 114-2. AmyR is the major activator of amylases and can repress the expression of cellulases. ClrB and XlnR are the major activators of cellulases and xylanases, respectively, and both can enhance the expression of cellulases. Deletion of *Pohtf1* led to a 117% increase in ClrB expression (Figure 7B), indicating that *Po*Htf1 might mediate the complex transcriptional-regulatory network cascade between developmental processes and cellulolytic gene expression mainly through regulating expression of ClrB in *P. oxalicum* 114-2.

Given the regulatory function of *Po*Htf1 in development and cellulase expression, *Po*Htf1 might act as a general regulator. To analyze the regulation patterns of *Po*Htf1 at the whole genome level, we performed differential transcriptome analysis of *P. oxalicum* 114-2 and Δ*Pohtf1*. The target genes regulated by *Po*Htf1 were widely distributed in the differentially expressed pathways, including starch and sucrose metabolism, glycolysis, and tyrosine metabolism (Supplementary Figure 1). In addition to the verified developmental regulator genes and cellulose hydrolyase genes, several secondary metabolism gene clusters were also influenced by *Po*Htf1 (Supplementary Table 3). These results revealed the general regulation role of *Po*Htf1 in the whole genome of *P. oxalicum* 114-2.

In this research, the functions of the homeodomain-containing protein *Po*Htf1 were characterized in *P. oxalicum* 114-2. *Po*Htf1 was not only crucial in colonial growth and conidiation but also an important transcriptional inhibitor of cellulases. Comparative transcriptome analysis also showed that *Po*Htf1 participated in several other metabolic pathways, including secondary metabolism.

## Data Availability Statement

The datasets presented in this study can be found in online repositories. The names of the repository/repositories and accession number(s) can be found in the article/Supplementary Material.

## Author Contributions

ZoL conceived and designed the experiments. RW, HG, GX, ZiL, MY, and ZJ performed the experiments. ZoL, MC, XB, and YQ analyzed the data. MC and ZoL drafted the manuscript. All authors read and approved the final manuscript.

## Conflict of Interest

The authors declare that the research was conducted in the absence of any commercial or financial relationships that could be construed as a potential conflict of interest.
