# Supplementary material for: A Homeodomain-Containing Transcriptional Factor PoHtf1 Regulated the Development and Cellulase Expression in Penicillium oxalicum
Source: Front Microbiol. 2021 Jun 10;12:671089. doi: 10.3389/fmicb.2021.671089 (PMC8222722; doi:10.3389/fmicb.2021.671089)
Supplement: Supplementary file 1 [file Data_Sheet_1.PDF]

Table S1. Primers used for construction of mutation strains

Primers	Primer Sequences（5'-3'）
Primers used for construction and verification of Δ*Pohtf1*	
ptra-F1	GGGCAATTGATTACGGGATC
ptra-R1	GCGGCTCATCGTCACCCCAT
7199-F1	TGCGATAGAAGCCGAGACCA
7199-ptraR	AATGGGATCCCGTAATCAATTGCCCGCAAATAGCGAGGAAGAACG
7199-ptraF	CAAGAGCGGCTCATCGTCACCCCATATTCGCACTGTGGGTATCTC
7199-R1	CAGAGCCTGGGCACTACGAG
7199-F2	CGGTACAACTGGCGGCTTTG
7199-R2	GCGTTCCCACTGCGTAAGAG
7199-yzF1	TGTTGTATGCCCTCAACTCG
7199-yzR1	ATCTGTCTATGGGTCATTCC
Primers used for construction and verification of C*Pohtf1*	
hph-F	CGACGTTAACTGATATTGAA
hph-R	CAACCCAGGGCTGGTGACGG
7199-hphF	GAAAATTCCGTCACCAGCCCTGGGTTGATCGGCATGGTCTCGCGATG
7199-hphR	GCTCCTTCAATATCAGTTAACGTCGTCAGTGAACCCTAAGACCAG
7199-F4	CCCTCGTAGCAACAGGATGT
7199-R4	CCTCGTCGGTGAAATGCTTC
hph-YZ-F	ATGAAAAAGCCTGAACTCAC
hph-YZ-R	CTATTCCTTTGCCCTCGGAC
ptrA-YZ-F	ATGTCTCCTCCAGCTGCCATC
ptrA-YZ-R	TTACTCAGCACACTCGCGC

Table S2. Primers used in qRT-PCR.

Primers	Primer Sequences（5'-3'）
RT-act-F	GTTCCATTCTCGCCTCCCTCT
RT-act-R	AGAAGCACTTGCGGTGAACGA
RT-cbh1-F	CCACCACCACTACCAGCAAGG
RT-cbh1-R	GTAGCCAACACCACCGCACT
RT-eg1-F	ACCGCTGCTCAGACCACGAC
RT-eg1-R	TGGGTCCCGAGTAGCCAACG
RT-bgl1-F	CACCAACACCGGCTCAGTTA
RT-bgl1-R	GGACATCCCAGTTGGACAGAT
RT-bgl2-F	GGCTGATGCGTACACGTTTGA
RT-bgl2-R	CGACATAAGTCACGCCGAAGC
RT-creA-F	ACAGTCCTGGTCAAGGTCAC
RT-creA-R	GCCCGCCACGGAATTATTTG
RT-clrB-F	TTGCCCGCATTTACGAAGCC
RT-clrB-R	GTCTTGGGGTCCATTTTCGC
RT-xlnR-F	GTGGTCCGAGCCTGCGAAAC
RT-xlnR-R	CAGCGGTAGAGGGCGAGAAC
RT-amyR-F	CCATCGGCAACTTTCTCCCA
RT-amyR-R	TGAATGCGCTCGTGATGCTC
RT-brlA-F	GGAACATCTCAAGCGGCACA
RT-brlA-R	CAACTTGGAGCCGTAGATGG
RT-fluC-F	GGTCTGCGACAAGCGGTTCA
RT-fluC-R	ACCCTCCTTCTCACCCTTGT
RT-stuA-F	GCGGCTCCTACACTTACACC
RT-stuA-R	GAACGGCATCGTCGTCATCG
RT-7199-F	CTCCACCAATGTCCGCACCA
RT-7199-R	ACGGTGACGCAGAGGCAGTA

Table S3. Transcription levels of secondary metabolism gene clusters regulated by *Po*Htf1.

cGene ID	Predected production/backbone genes	Transcription level (RPKM, Reads Per Kb per Million reads)						Change
		114-2 (1)	114-2 (2)	114-2 (3)	Δ*Pohtf1* (1)	Δ*Pohtf1* (2)	Δ*Pohtf1* (3)	
**Cluster_1**								
PDE_00787		48.91	47.66	41.67	42.62	41.68	37.62	
PDE_00788		3.64	2.44	2.29	3.46	2.08	2.64	
PDE_00789	NRPS	23.61	29.91	34.17	1.36	1.15	1.63	↓
PDE_00790		64.74	86.46	79.59	2.32	1.34	4.04	↓
PDE_00791		36.45	46.75	44.30	21.74	25.06	25.64	↓
PDE_00792		51.37	68.45	68.30	1.04	0.58	0.94	↓
PDE_00793	NRPS	11.94	13.72	14.57	3.07	3.40	3.01	↓
PDE_00794		74.51	86.61	81.66	1.51	0.74	2.52	↓
PDE_00795		16.99	21.94	20.33	0.32	0.08	1.27	↓
PDE_00796		20.12	28.65	23.32				↓
PDE_00797	DMAT	102.99	124.65	118.83	2.32	2.36	9.96	↓
PDE_00798		80.27	112.41	97.37	3.15	1.90	8.31	↓
PDE_00799		54.27	55.79	58.79	4.11	3.44	7.88	↓
PDE_00800		16.95	23.05	16.62	1.50	1.30	2.89	↓
PDE_00801		13.47	15.73	11.92	1.56	1.29	2.40	↓
PDE_00802		12.54	18.43	17.22	1.19	1.38	2.97	↓
PDE_00803		17.74	27.84	20.89	0.24			↓
PDE_00804		20.36	27.60	25.64	0.25	0.12	0.66	↓
PDE_00805		33.47	45.31	41.62	0.13	0.26	0.28	↓
PDE_00806		43.20	57.61	53.37	0.99	0.36	0.52	↓
PDE_00807	DMAT	22.81	29.59	25.53	0.10			↓
PDE_00808		22.89	28.86	26.85	0.39	0.31	1.07	↓
PDE_00809		11.43	10.95	11.04				↓
PDE_00810	NRPS	5.42	4.94	4.92	1.51	1.92	1.89	↓
PDE_00811		35.92	40.16	36.50	1.72	0.96	2.59	↓
PDE_00812		14.52	6.58	9.21	5.07	4.74	8.02	
PDE_00813		0.44	0.77	1.24	0.21	0.62	0.44	
PDE_00814		12.97	11.01	17.43	10.40	10.89	13.20	
**Cluster_2**	**Oxaline**							
PDE_01064		9.89	12.32	11.72	0.16	0.23	0.59	↓
PDE_01065		73.98	90.58	79.55	1.25	0.68	3.82	↓
PDE_01066	DMAT	125.71	132.47	127.56	3.73	2.67	9.03	↓
PDE_01067		4.45	6.32	6.21	1.06	0.89	0.88	↓
PDE_01068		34.49	38.96	38.45	2.48	3.57	4.96	↓
PDE_01069		31.93	35.15	25.09	0.71	0.79	2.24	↓
PDE_01070		5.43	6.65	6.79	0.17	0.08	0.27	↓
PDE_01071	NRPS	0.65	1.13	0.84	0.07	0.03	0.09	↓

PDE_01072		-	-	-				↓
PDE_01073		33.95	50.82	37.65	0.44	0.11	1.74	↓
PDE_01074		9.91	12.39	10.19	0.08			↓
PDE_01075		0.92	1.04	0.71				↓
PDE_01076		0.97	1.61	1.18	0.08			↓
PDE_01077	NRPS	0.48	0.58	0.63	0.13	0.13	0.27	↓
**Cluster_5**								
PDE_01202		2.03	1.80	1.35	24.76	7.24	4.67	
PDE_01203		0.17	-	-				
PDE_01204		-	-	-				
PDE_01205		0.89	0.60	0.92	0.28	0.55	0.30	
PDE_01206		36.44	39.20	45.15	29.96	44.07	47.81	
PDE_01207		48.11	50.70	54.17	21.14	31.79	34.27	
PDE_01208		68.87	67.98	85.06	25.12	35.26	50.32	
PDE_01209		0.86	0.87	0.89	0.72	0.70	0.32	
PDE_01210		22.71	37.46	43.49	14.91	20.10	21.62	
PDE_01211		60.04	70.83	77.63	41.70	59.49	63.08	
PDE_01212	NRPS	167.96	206.10	135.52	213.48	249.94	196.61	↑
PDE_01213		315.70	402.93	242.78	705.90	548.31	553.49	↑
PDE_01214		367.95	478.16	282.98	716.63	532.25	611.56	↑
PDE_01215		1666.55	2099.58	1324.08	4124.80	3371.99	3392.53	↑
PDE_01216		979.78	1202.36	792.19	2179.76	1897.28	2040.16	↑
PDE_01217		456.60	626.67	396.09	805.31	763.24	811.51	↑
PDE_01218		422.65	570.90	397.60	598.67	701.35	807.39	↑
**Cluster_7**								
PDE_01418		236.41	227.48	224.62	118.94	120.53	129.61	
PDE_01419		9.64	9.82	9.73	10.00	10.57	7.28	
PDE_01420		3.62	5.59	4.83	4.77	5.83	6.19	
PDE_01421		10.28	11.57	12.74	10.50	15.10	12.59	
PDE_01422		14.15	17.72	21.65	7.32	8.83	19.26	
PDE_01423		0.41	0.41	0.42				
PDE_01424		166.18	208.19	193.46	170.76	165.11	211.28	
PDE_01425		4.70	5.56	3.44	3.79	4.48	2.54	
PDE_01426		0.78	1.18	1.20	0.52	0.95	0.78	
PDE_01427		0.90	0.72	1.20	0.34	0.42	0.81	
PDE_01428		0.77	1.55	1.98	1.35	1.44	0.65	
PDE_01429		1.65	1.96	2.70				↓
PDE_01430		3.90	6.39	5.77				↓
PDE_01431		18.10	21.17	15.92	1.53	2.32	2.78	↓
PDE_01432	NRPS	4.98	5.76	3.90	0.28	0.34	0.47	↓
PDE_01433		6.85	9.14	6.96				↓
**Cluster_8**								

PDE_02130		0.39	0.91	0.36	0.34	1.17	0.49	
PDE_02131	NRPS	1.59	2.13	2.39	0.99	1.62	1.28	
PDE_02132		18.87	20.37	20.68	16.19	21.32	15.95	
PDE_02133		27.87	34.73	28.26	24.02	37.86	25.59	
PDE_02134		0.59	0.84	0.49	4.74	13.73	3.33	
PDE_02135		0.27	0.30	0.36	0.18	0.78	0.27	
PDE_02136		11.92	11.10	10.81	13.42	16.15	13.25	
PDE_02137		0.92	1.31	1.20	1.11	1.91	0.88	
PDE_02138		24.59	29.45	26.64	2.08	1.09	1.75	↓
PDE_02139		32.05	36.92	35.84	1.56	0.92	3.14	↓
PDE_02140		43.31	51.41	48.03	6.68	3.39	7.53	↓
PDE_02141	PKS	10.63	17.92	16.09	0.53	0.60	1.26	↓
**Cluster_13**								
PDE_03453		1.86	3.38	2.73	1.43	0.83	0.71	↓
PDE_03454		37.27	31.69	35.08	6.02	4.27	7.23	↓
PDE_03455	PKS	7.14	8.39	10.01	1.74	2.86	3.40	↓
**Cluster_14**								
PDE_03920		203.72	221.41	222.31	272.29	291.45	282.92	
PDE_03921		53.54	60.07	59.31	36.59	45.00	45.39	
PDE_03922		0.60	0.80	0.41	0.57	0.56	1.00	
PDE_03923		-	-	0.16	0.29			
PDE_03924		1.56	1.85	2.77	1.48	1.59	1.42	
PDE_03925		46.73	53.80	54.10	39.34	42.17	58.04	
PDE_03926	PKS	3.95	4.81	4.13	18.36	14.18	3.73	↑
PDE_03927		171.71	187.77	212.19	500.28	414.13	466.64	↑
PDE_03928		52.46	59.30	57.50	260.88	171.79	129.86	↑
PDE_03929		4.45	6.61	6.67	16.37	11.63	8.19	↑
**Cluster_25**	**Aspyridones**							
PDE_09188		0.39	0.49	0.61	0.37	0.55	0.69	
PDE_09189		0.64	0.86	1.54	1.22	1.60	0.75	
PDE_09190		1.83	1.41	1.55	2.15	3.01	1.62	
PDE_09191		-	0.28	-				
PDE_09192		2217.77	2644.44	1664.64	6.39	3.01	0.94	↓
PDE_09193		91.03	120.43	85.19	3.30	2.95	3.33	↓
PDE_09194		137.27	184.26	134.13	41.28	51.04	43.33	↓
PDE_09195		930.26	1004.53	940.86	996.81	1034.72	919.78	↓
PDE_09196		234.88	342.99	216.86	7.96	10.25	5.56	↓
PDE_09197		11.15	10.74	6.88	11.87	10.54	2.87	↓
PDE_09198	HYBRID	6.62	11.48	7.47	1.49	1.72	1.81	↓
PDE_09199		52.00	76.01	52.46	0.08	0.15	0.17	↓
PDE_09200		27.88	40.14	21.92	0.76	1.02	1.39	↓
PDE_09201		33.36	42.03	24.71	0.35	0.57	1.10	↓

PDE_09202		9.03	14.49	9.83				↓
**Cluster_26**	**Conidial yellow pigment**							
PDE_09227		34.21	34.24	36.57	24.00	18.91	26.38	
PDE_09228		1.64	1.42	1.53	1.98	1.32	1.57	
PDE_09229		4.59	4.88	4.03	1.51	2.10	2.35	↓
PDE_09230		19.45	22.70	19.40	7.87	9.58	9.89	↓
PDE_09231		3.71	4.39	4.48	1.84	1.36	2.27	↓
PDE_09232		0.33	0.33	-				↓
PDE_09233		0.48	0.67	0.29				↓
PDE_09234		2.95	1.48	0.34	0.16	0.15	0.16	↓
PDE_09235		1.19	1.54	0.70	0.16	0.16	0.85	↓
PDE_09236		0.74	3.22	1.77				↓
PDE_09237	PKS	0.19	0.39	0.07	0.05			↓
PDE_09238		0.14	-	-				↓
PDE_09239		7.93	6.36	5.33	1.70	0.45	3.25	↓
PDE_09240		2.80	2.53	2.30	0.53	0.26	1.12	↓
PDE_09241		2.06	2.08	3.64				↓
PDE_09242		57.38	64.36	69.45	31.47	36.06	137.09	↓
PDE_09243		52.86	54.99	54.16	30.31	30.96	48.97	↓
**Cluster_28**								
PDE_09994		197.49	215.98	250.88	239.73	220.46	344.18	
PDE_09995		1.87	1.62	1.93	0.64	0.50	0.94	
PDE_09996		2.87	2.89	4.03	2.36	4.26	2.75	
PDE_09997		-	-	-	0.33			
PDE_09998		-	-	-				
PDE_09999		8.13	12.11	10.41	0.33	0.08	0.26	↓
PDE_10000		54.29	62.87	62.35	2.93	2.70	7.27	↓
PDE_10001		24.09	27.05	27.78	1.11	0.55	2.64	↓
PDE_10002		31.80	41.54	36.12	1.26	1.08	4.49	↓
PDE_10003		35.24	51.00	44.04	0.71	0.77	2.23	↓
PDE_10004		6.27	7.34	9.07	0.56	0.71	0.51	↓
PDE_10005		19.02	24.71	22.21	0.35	0.69	1.11	↓
PDE_10006	PKS	1.66	1.98	2.04	0.12	0.03	0.11	↓
PDE_10007		21.78	28.40	29.36	2.72	1.76	3.92	↓
PDE_10008		6.98	9.21	7.10	0.79	0.31	2.09	↓
PDE_10009		20.80	25.70	27.91	0.98	0.14	1.48	↓

Figure S1

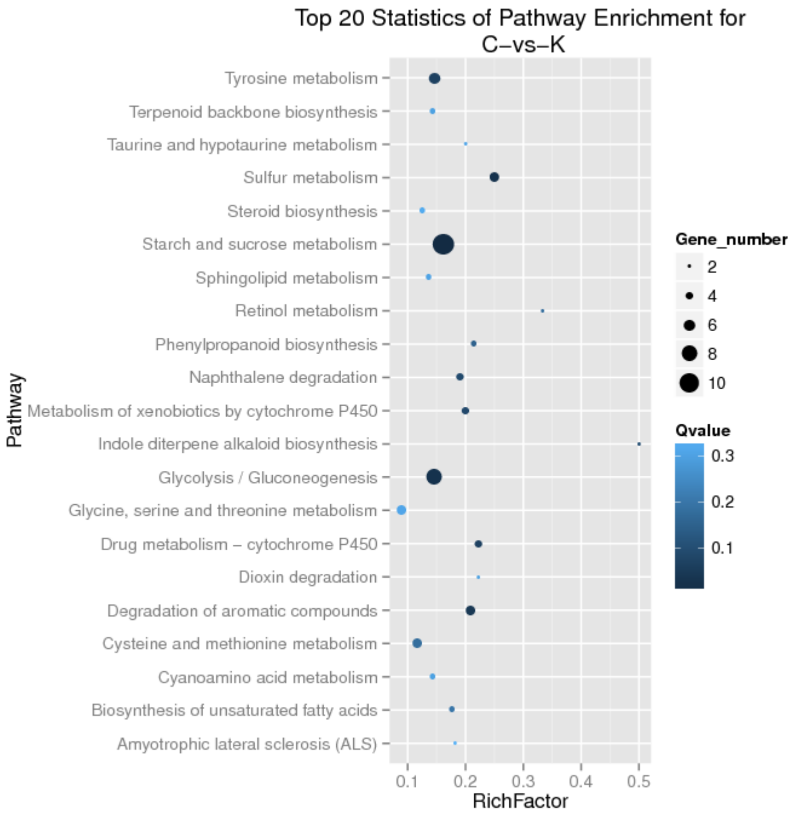

Figure S1. Pathway enrichment of the differentially expressed genes in *P. oxalicum* 114-2 and Δ*Pohtf1*. Sample C stands for *P. oxalicum* 114-2, K stands for Δ*Pohtf1*.

Figure S2

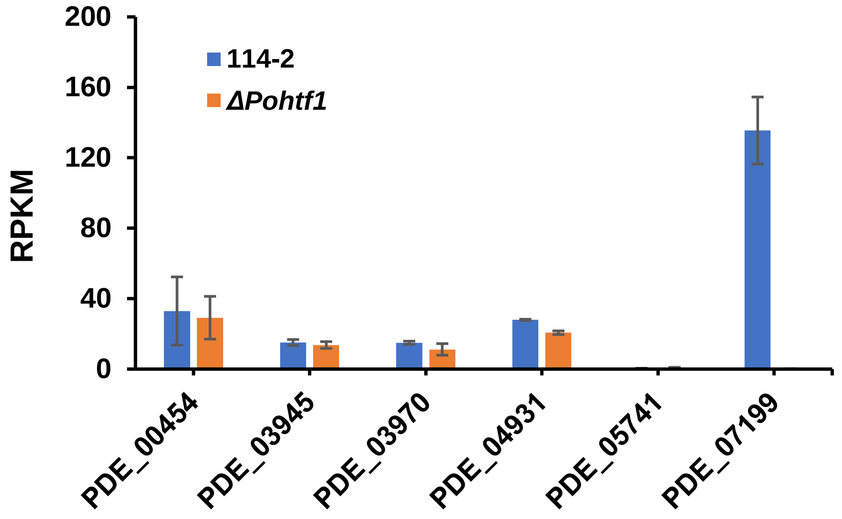

Figure S2. The transcriptional levels of 6 PoHtfs in *P. oxalicum* 114-2 and Δ*Pohtf1*. The average RPKMs of 6 PoHtfs in *P. oxalicum* 114-2 and Δ*Pohtf1*.

Figure S3

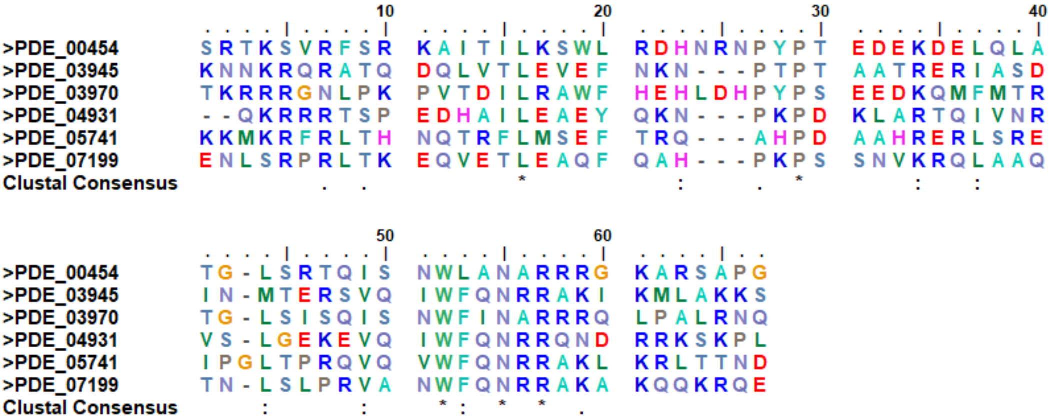

Figure S3. Sequence alignment of homeodomains of six *Po*Htfs in *P. oxalicum* 114-2. The homeodomain of *Po*Htfs were analyzed by the Simple Modular Architecture Research Tool (SMART). The alignment was performed by ClustalW Multiple Alignment function in Bioedit tool. The consensus residues were labeled by asterisk (*).
